# Neuroblastoma Interaction with the Tumour Microenvironment and Its Implications for Treatment and Disease Progression

**DOI:** 10.3390/curroncol30100659

**Published:** 2023-10-15

**Authors:** Leila Jahangiri

**Affiliations:** 1 School of Science and Technology, Nottingham Trent University, Clifton Site, Nottingham NG11 8NS, UK; leila.jahangiri@ntu.ac.uk; 2 Division of Cellular and Molecular Pathology, Addenbrookes Hospital, University of Cambridge, Cambridge CB2 0QQ, UK

**Keywords:** neuroblastoma, tumour microenvironment, disease progression, treatment

## Abstract

Neuroblastoma, a paediatric malignancy of the peripheral nervous system, displays a wide range of clinical outcomes, including regression to fatality despite extensive treatment. Neuroblastoma tumours display a complex interplay with their surrounding environment, known as the tumour microenvironment, which may affect disease progression and patient prognosis. This study aimed to dissect the ways in which neuroblastoma biology, treatment, prognosis, progression, and relapse are linked with the extracellular matrix, the dichotomous identities of neuroblastoma, various regulatory proteins and RNA, and extracellular vesicles within the backdrop of the tumour microenvironment. In addition, other aspects, such as immune cell infiltration, therapeutic options including monoclonal antibodies and small molecule inhibitors; and the ways in which these may affect disease progression and immunosuppression within the context of the neuroblastoma tumour microenvironment, are addressed. Such studies may shed light on useful therapeutic targets within the tumour microenvironment that may benefit groups of NB patients. Ultimately, a detailed understanding of these aspects will enable the neuroblastoma scientific community to improve treatment options, patient outcomes, and quality of life.

## 1. Introduction to NB and the Tumour Microenvironment

Neuroblastoma (NB) is a rare heterogeneous paediatric tumour that, despite only accounting for circa 10% of cancer diagnoses in children, disproportionately leads to 15% of cancer-related deaths in this age group [1,2]. Circa 1200 new cases of NB are diagnosed per annum in the United States and Europe [1,3]. Further, NB is a cancer of the sympathetic nervous system presenting in the abdominal cavity, specifically in the adrenal glands and sympathetic ganglia along paravertebral sympathetic chains [4]. These sympathetic chains and suprarenal ganglia originate from the neural crest cells [5], while single-cell sequencing suggests that neural crest-like Schwann cell precursors (SCPs) give rise to sympathoblasts, which in turn transition to chromaffin cells [6]. Due to the neuroendocrine nature of NB cells, they secrete catecholamines, which may activate β-adrenergic receptors [7].

The staging of NB was initially developed by the International Neuroblastoma Staging System (INSS) based on the propensity for surgical removal of the tumour; therefore, stages 1 and 2 encompassed localised tumours with complete and incomplete gross excision potential, respectively [8]. Stage 3 represented the unilateral presence of a tumour across the midline in the absence or presence of lymph node involvement, and stage 4 represented any primary tumour with dissemination to distant tissue. Finally, 4S represented localised primary tumours limited to bone marrow (not cortical bone), liver, and skin in patients under the age of 12 months [8]. INSS may not be suitable for pretreatment stratification; hence, the International Neuroblastoma Risk Group (INRG) defined the INRG staging system (INRGSS). In this classification system, locoregional tumours are classified as L1 and L2 on the grounds of the absence or presence of image-defined risk factors (IDRFs), respectively. IDRFs are specific features within images that are linked to a poor prognosis. Further, metastatic tumours are referred to as M except for those that are metastatic special (MS), in which there is a confinement of metastasis to specific tissues such as the bone marrow (not cortical bone), skin, and liver in patients younger than the age of 18 months [9]. In addition, risk stratification at the time of diagnosis is based on MYCN amplification status (an MYCN copy number increase is strongly linked to poor prognosis), ploidy, stage, age, and segmental chromosomal alteration status (e.g., 11q loss), and it is noteworthy that close to half of all cases of NB are categorised as high-risk and only half of these cases achieve long-term survival [4,9,10]. Many high-risk NB tumours initially respond to treatment inclusive of COJEC (cisplatin, vincristine, carboplatin, etoposide, and cyclophosphamide) within 70 days on 10-day intervals (8 in total), followed by stem cell transplantation, surgery, radiotherapy, isotretinoin, and anti-GD2 antibodies to eliminate any residual disease. Most NB patients display remission; however, relapse may still occur due to the dominance of treatment-resistant clones [11,12].

**Figure 1:**
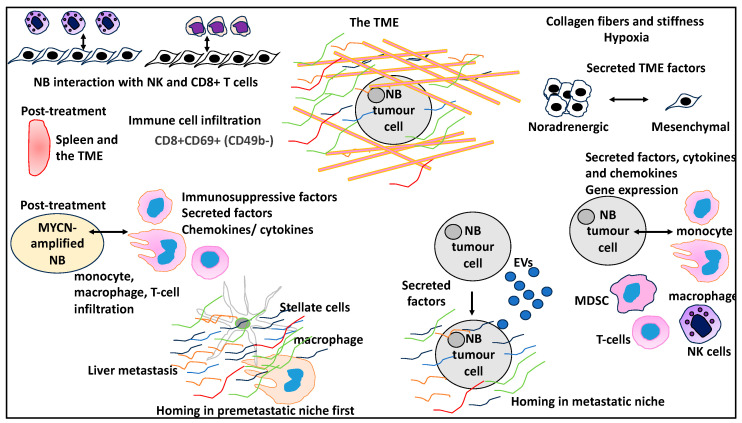
A summary of the molecular structures, secreted factors, and immune and stromal cells that interact with NB tumour cells and may impact NB biology, prognosis, treatment, relapse, and progression. ECM aspects such as collagen fibres, stiffness, and hypoxia will impact NB tumours. Secreted factors in the TME can impact the inherent states of NB cells and trigger the transition from noradrenergic to mesenchymal states. NB cells can interact with a myriad of immune cells, such as monocytes, macrophages, MDSCs, T cells, and NK cells, and changes to gene expression and the secretion of cytokines, chemokines, and other factors may mediate these interactions. NB cells can secrete EVs that contain proteins that may prime the TME, including a secondary niche, and facilitate NB cell homing and metastasis. Similarly, in secondary metastasis sites such as the liver, immune and stromal cells, including macrophages and hepatic stellate cells, may initially prime the secondary TME for the entry of NB tumour cells at a later time. In addition, NB samples post-treatment may show infiltration of immune cells such as monocytes, macrophages, and T cells, and a myriad of secreted factors, chemokines, and cytokines in the TME could induce immunosuppression. The post-treatment spleens and the TME of the treatment recipient may also be a site for immune cell infiltration, including CD8+ T cells. Finally, the interaction between primed NK cells and NB cells may lead to reduced NB tumour size, while the blockage of CD8+ T cell-targeting suppressive proteins can lead to a higher level of CD8+ T-cell activation and antitumour effects.

NBs are highly heterogeneous, which may reflect their divergent molecular and clinical attributes, leading to a variable clinical course and treatment efficacy [11,12,13,14,15]. Cellular and molecular mechanisms, various molecules, and processes of the tumour microenvironment (TME) may contribute to this observed heterogeneity [16]. For example, the TME, which includes the extracellular matrix (ECM), is a highly dynamic network comprising numerous proteoglycans, proteins, and glycoproteins that constantly undergo deposition and degradation, allowing for the instigation and integration of signals that may affect the NB tumour and its biological and clinical attributes [17,18]. Furthermore, the intrinsic properties of NB tumours and their profile of differentially expressed RNA and proteins may contribute to the interaction between NB and the TME [19,20,21]. The TME also includes stromal and immune cells and a repertoire of secreted molecules, cytokines, and chemokines [15]. Concerning the immune landscape within the TME in NB, recent studies showed the presence of T cells, B cells, natural killer (NK) cells, and myeloid cells such as myeloid-derived suppressor cells (MDSCs) [22]. NB tumours may also differ in their tendency to harbour various types of immune cells, including CD8+ T cells or noninflammatory M2 macrophages [23]. Moreover, extracellular vesicles (EVs), which are heterogenous vesicles varying in size from apoptotic bodies and large EVs to small EVs, contain proteins, RNA, and lipids and can be produced by tumours to influence stromal and immune cells within the TME [24]. This will, per se, impact key cancer-related processes such as tumour proliferation, survival, immune escape, and resistance to treatment [25,26]. For example, EVs may deliver key factors such as IGF2BP1 to the TME to promote ECM protein deposition and the recruitment of immune cells to prime metastatic organs, thereby promoting disease progression in NB [21]. Finally, small molecule inhibitors and monoclonal-antibody-based treatments can also impact the TME [27], while the blockage of immunosuppressive ligands may also improve T-cell antitumour responses [28] (Figure 1).

Given this background, this study aimed to understand the role of various molecular and cellular processes, molecules, and players in the NB TME and how they may affect NB biology, treatment, prognosis, progression, and relapse.

## 2. The TME in NB and Its Link to NB Biology and Clinical Aspects

### 2.1. Immune and Stromal Landscapes of NB TME

Before delving into the topic of the interaction of NB tumour cells with the TME from the viewpoint of various aspects, the immune and stromal cell landscape of the TME will be addressed in this section. Apart from the immune cells mentioned in the introduction [22,23], the NB TME may also harbour other immune and stromal cells, such as macrophages and fibroblasts. A study investigated 41 NB patient samples for the presence of various immune and stromal cells, and they found the presence of tumour-associated macrophages (TAMs) that were positive for CD68, CD163, and CD204, in addition to the presence of cancer-associated fibroblasts (CAFs) that were positive for α smooth muscle actin (α-SMA) [29]. Interestingly, the number of TAMs and the surface of CAFs were linked to various clinicopathological factors such as stage, MYCN status, and metastasis. For example, the number of CD68/CD163-expressing macrophages was linked to aggressive phenotypes, including MYCN amplification [29]. In addition, the authors suggested that the CAFs and TAMs resided nearby, indicative of interaction with the TME. The authors also tested whether the conditional medium of NB (supernatants obtained from culturing NB cells) could induce TAM markers in peripheral blood mononuclear cell (PBMC)-derived macrophages, and it was shown that CD163 and CD204 were upregulated in macrophages obtained this way [29]. Likewise, the NB conditional medium included α-SMA in bone marrow-derived mesenchymal stem cells (BM-MSCs) to form fibroblasts. Finally, secreted molecules such as CXCL2 from these TAMs increased tumour invasiveness. In conclusion, the interesting study not only outlined the presence of TAMs and CAFs in the NB TME but also delicately deciphered their interactions with the tumour cells. BM-MSCs and PBMCs were recruited to the TME and, due to the influence of NB tumour cells, gave rise to CAFs and TAMs, respectively [29].

In agreement with this study, another study utilised the power and resolution of single-cell transcriptomics to better understand the TME of NB in a MYCN-driven mouse model and patient primary tissue [30]. The authors revealed that MYCN-driven mouse tumours are low in T cells but higher in macrophage, MSDC, and CAF content, corresponding with an overall immunosuppressive environment [30]. A similar observation was made with the patient tissue in that an intricate network of myeloid cells was detected. T cells in the mouse model and patient primary tissue showed inhibitory receptor expression and exhaustion phenotypes, respectively. The former may indeed be due to the suppressive effects of MDSCs on T cells [30]. Another study also reported the presence of Treg, MDSCs, and macrophages and a low content of CD8+ T cells in MYCN-amplified NB samples [31]. Apart from the type of immune and stromal cells that may interact with NB cells, these cells may produce components of the ECM that encourage tumourigenesis [32]. The authors of a study examined collagen expression using the COL11A1 marker in NB primary tissue, and it was shown that NB cells and SMA+ CAFs upregulated this gene. The coculture of CAFs and NB cells also led to increased NB cell invasion, while the downregulation of COL11A1 expression in CAFs reduced NB invasion, suggesting that immune and stromal cells of the TME might also contribute by affecting the ECM protein content, and CAFs can prime NB cells [32]. In conclusion, these four studies confirmed the general immunosuppressed ecosystem of NB TME and therefore emphasised the complex work ahead for targeting and manipulating this TME.

Given this background, in the following subsections, studies that addressed the targeting and manipulating of various aspects of the TME from the lens of understanding and improving NB biology, treatment, prognosis, progression, and relapse will be reviewed.

### 2.2. The Physical Properties of the ECM within the TME That Are Linked to NB Biology

NB tumours may be affected by the ECM based on biochemical and physical interactions; for example, biophysical properties such as stiffness can affect NB proliferation and neuritogenesis. In evidence of this, the retinoic acid (RA)-mediated NB differentiation of SK-N-DZ cells was affected by mechanical stiffness (e.g., culture in a 1000 KPa collagen-coated polyacrylamide gel). The combination of stiffness and RA treatment enhanced NB differentiation and reduced proliferation and tumour markers such as MYCN (a gene linked to NB high-risk prognosis) [33]. The study suggested that the biophysical aspects of the ECM also affected NB differentiation.

In a similar study, collagen, as a major component of the TME, was found to be vulnerable to thermal ablation [34]. To that end, the authors of a study used SK-N-DZ spheres to thermally ablate collagen using a fibre-coupled diode laser beam, while the native collagen was detected using an anticollagen 1 antibody, and the degraded collagen was detected using FAM conjugated to collagen hybridising peptides (Fab-CHP) [34]. In evidence of this, thermal ablation led to the loss of detection of col1 protein and the detection of Fab-CHP. This treatment also led to a 2.8-fold decrease in the proliferation rate of these NB cells and reduced migration and TME stiffness. From a mechanistic viewpoint, collagen denaturation reduced LOX/LOXL2 expression levels and decreased focal adhesion kinase (FAK) phosphorylation [34]. Downstream of reduced FAK phosphorylation, the authors observed reduced epithelial-to-mesenchymal transition (assessed by reduced N-cadherin and vimentin levels) and reduced migration (assessed by reduced CDC42 levels). In conclusion, collagen is a key component of the ECM, and it is linked to the LOX/LOXL2-FAK pathway [34]. Alterations to the biophysical properties of the ECM, such as the thermal ablation of collagen, lead to changes in various signalling pathways and therefore may be an important aspect to consider for in vivo studies.

**Figure 2:**
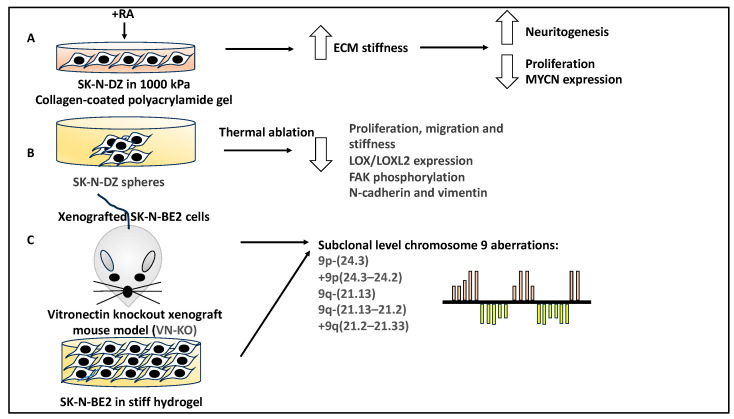
The role of the biophysical properties of the ECM on NB biology. (**A**) RA-based NB differentiation of SK-N-DZ cells cultured in the presence of collagen-coated polyacrylamide gel asserting a stiffness of 1000 KPa. This treatment led to enhanced neuritogenesis in addition to reduced proliferation and MYCN expression. (**B**) SK-N-DZ spheres were treated with thermal ablation to denature the TME collagen, and this led to reduced proliferation, migration, and stiffness, LOX/LOXL2 expression, FAK phosphorylation, and reduced N-cadherin and vimentin levels. (**C**) SK-N-BE2 cells xenografted to a vitronectin knockout mouse model (VN-KO) and SK-N-BE2 cells cultured in rigid and stiff hydrogels for an extended period revealed (focal) segmental chromosomal alterations, including (9p-(24.3), +9p(24.3–24.2), 9q-(21.13), 9q-(21.13–21.2), and (+9q(21.2–21.33)). SH-SY5Y cells used as a control for both readouts showed genomic stability.

A study reported that various glycoproteins, such as vitronectin, were elevated in high-risk NB. Interestingly, using a vitronectin knockout xenograft mouse model, a study detected a subclonal genomic alteration in MYCN-amplified SK-N-BE2 cells grown in these mice. This was also observed in SK-N-BE2 cells cultured in stiff hydrogels for extended periods. Specifically, subclones with chromosome 9 alterations were detected in both systems, suggesting the role of biophysical parameters in affecting NB genetic alterations consistent with tumour evolution [35]. Some of the common genetic alterations detected for chromosome 9 in both methods included segmental chromosomal alterations of 9q-(21.13) and 9q-(21.13–21.2)). This study also utilised the SH-SY5Y NB cell line as a control, which remained genetically stable under both conditions [35]. In conclusion, this study outlined the link between the ECM properties within the TME and NB genomics and prognosis in both cell culture and animal models that could be further manipulated for improving NB treatment outcomes and patient prognosis (Figure 2).

### 2.3. NB Mesenchymal to Noradrenergic Identity Transition Is Influenced by the TME

Studies by Van Groningen and colleagues [36,37] identified noradrenergic and mesenchymal identities established by core regulatory circuitries (CRCs), which comprise tightly regulated superenhancer-driven transcription factors that self-regulate and regulate each other and a network of target genes [38,39]. Noradrenergic or mesenchymal identities have been linked to CRCs, including PHOX2A/B and HAND1/2, or the AP1 family, respectively [36,37]. Using single-cell RNA sequencing, the study attempted to understand the role of the TME and external cues on NB identity in the SK-N-SH, SH-EP, and SH-SY5Y and patient-derived xenografts (PDXs) [19].

Using single-cell transcriptomics, the authors revealed that CD44 was the marker of mesenchymal identity, with CD44− and CD44+ representing SH-SY5Y (noradrenergic) and SH-EP (mesenchymal) cell lines, respectively (both cell lines were originally derived from the parental SK-N-SH cell line) [19].

Cell lines were then grown from 15 PDXs, and, for example, IC-pPDXC-63 grew biphenotypically, containing both adherent cells and spheres, representing mesenchymal (CD44+) and noradrenergic (CD44−) identities, respectively, while single-cell RNA sequencing of this PDX-derived cell line also showed noradrenergic and mesenchymal clusters with a bridge population [19]. Interestingly, the CD44+ FACS-sorted cells and adherent cells showed similar transcriptomic profiles to SH-EP cells (mesenchymal), while CD44− cells and spheres clustered with noradrenergic cells such as SH-SY5Y cells [19].

The authors then explored the plasticity of NB cells, including IC-pPDXC-63 CD44− (noradrenergic) and CD44+ (mesenchymal), by subjecting the cells to FACS and separate culturing. It was shown that within the IC-pPDXC-63 population, only the CD44− (noradrenergic) cells gave rise to both populations of CD44− and CD44+ cells [19].

The authors then attempted to characterise the role of extrinsic factors of the TME that influenced the noradrenergic-to-mesenchymal transition (NMT) and showed that *EGFR* and *TNFRSF1A* (the receptors for EGF and TNF-α, respectively) were expressed in the mesenchymal fraction of IC-pPDXC-63 cells and the bridge population. Subsequently, with SK-N-SH noradrenergic/CD44− cells, EGF and TNF-α treatment for three days in culture prompted NMT, decreasing the PHOX2B+/CD44− population and increasing the CD44+ fraction, suggesting the effect of extrinsic factors on NMT that influenced NB identities within the simulated TME [19].

Further, SK-N-SH CD44− FACS-sorted cells and IC-pPDXC-63 floating cells were studied by ChIP sequencing for CRCs, and it was shown that they displayed a noradrenergic phenotype (group I linked to *GATA3*, *PHOX2B*, and *HAND1* genes). The IC-pPDXC-63 adherent cells and SK-N-SH CD44+ FACS-sorted cells displayed a mesenchymal phenotype (group II linked to *RUNX1*, *FOSL1*, and *TBX18* genes) [19].

Finally, noradrenergic/CD44− and mesenchymal/CD44+ cells isolated from IC-pPDXC-63 and SK-N-SH were xenografted to nude mouse models and all formed tumours in vivo [19]. Upon performing immunohistochemistry (IHC) on the tumours obtained from xenografting, it was revealed that *PHOX2B* was expressed in the majority of these tumours, even those originating from mesenchymal cell populations. From a CRC viewpoint, the epigenetic landscape of the xenografted tumours (e.g., originating from SK-N-SH CD44+ and CD44− cells) showed a noradrenergic phenotype (group I), suggesting that in vivo cues induced transition to adrenergic status [19].

**Figure 3:**
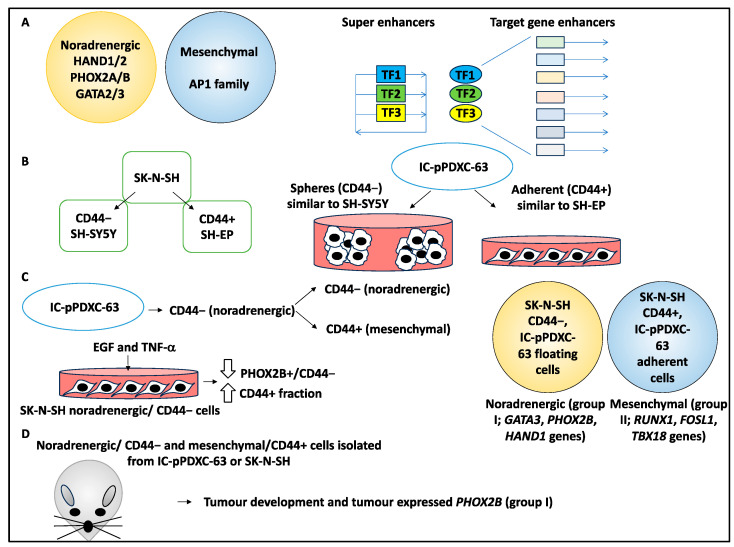
Mesenchymal and noradrenergic phenotypes and their links to NB biology. (**A**) Previous studies deciphered two identities, inclusive of noradrenergic and mesenchymal, defined by CRCs. As shown in the figure, CRC transcription factors (TFs) driven by superenhancers self-regulate and regulate each other. These TFs bind to the regulatory regions of their target genes and drive a specific identity. Noradrenergic CRCs include PHOX2A/B, GATA2/3, and HAND1/2, while mesenchymal CRCs include the AP1 family. (**B**) SK-N-SH is the parental cell line of SH-EP and SH-SY5Y cells. Using single-cell transcriptomics, it was shown that the CD44− mark identified SH-SY5Y and the CD44+ mark identified SH-EP cells. PDX-derived cell lines such as IC-pPDXC-63 grew biphenotypically, containing both adherent cells and spheres, representing mesenchymal and noradrenergic identities, respectively. Single-cell RNA sequencing showed CD44+ sorted cells and adherent cells displayed a similar identity to SH-EP cells, while CD44− and spheres were similar to adrenergic SH-SY5Y cells. (**C**) IC-pPDXC-63 cells were FACS-sorted into CD44− (noradrenergic) and CD44+ (mesenchymal) cells, and only CD44− (noradrenergic) cells gave rise to a heterogeneous population of CD44− and CD44+ cells. SK-N-SH noradrenergic/CD44− cells were treated with EGF and TNF-α for three days in culture, this decreased the PHOX2B+/CD44− population and increased the CD44+ fraction, allowing for NMT. SK-N-SH CD44- FACS-sorted cells and IC-pPDXC-63 floating cells displayed noradrenergic phenotypes (group I; *GATA3*, *PHOX2B*, *HAND1* genes) assayed by ChIP-sequencing of CRCs. The IC-pPDXC-63 adherent cells and SK-N-SH CD44+ FACS-sorted cells displayed a mesenchymal phenotype (group II; *RUNX1*, *FOSL1*, *TBX18* genes). (**D**) Noradrenergic/CD44− and mesenchymal/CD44+ cells isolated from IC-pPDXC-63 and SK-N-SH were xenografted to nude mouse models, and all formed tumours and predominantly showed noradrenergic phenotypes.

In conclusion, this study examined the fluidity of NB identities and, most importantly, found that noradrenergic cells in vitro, under the influence of strong cues from the TME, such as inflammatory mediators and growth factors, transitioned to the mesenchymal status. In contrast, in vivo, strong cues favoured the noradrenergic status, revealing a disparity in outcomes based on the nature of the TME that ultimately induced a transition in NB cell status and may impact NB progression and prognosis (Figure 3).

### 2.4. The Role of Differential Expression and Various Proteins in Influencing the TME in NB through Cytokines and Chemokines

The role of monocytes, TAMs, and alternative splicing events in the NB TME were addressed [20,29]. The authors initially investigated polypyrimidine tract binding protein 2 (PTBP2), and PTBP2 was detected in the SK-N-SH cell line, but knocking down this gene did not affect its viability, instead affecting its migration potential [20].

The authors studied the supernatant of NB cells using cytokine assays following PTBP2 modulation, and it was shown that CCL5 was altered (a cytokine that regulates monocytes, TAMs, and regulatory T-cell recruitment to the microenvironment). Accordingly, CCL5 was a target of PTBP2 since it was up- and downregulated following the overexpression and knockdown of PTBP2, respectively. In addition, the conditional medium of PTBP2-altered SK-N-SH cells was collected and used to treat monocytes and macrophages and it was shown that this alteration could affect the chemotactic activity of monocytes and macrophages [20]. Accordingly, the overexpression of PTBP2 and its downregulation increased and decreased monocyte and macrophage chemotaxis, respectively, indicating that in the TME, NB cells can impact the movement of immune cells. Likewise, PTBP2 knockdown decreased CD14+ CCR5+ monocyte cell presence [20].

Additionally, antibody-mediated blocking of CCL5 in PTBP2-expressing NB cells inhibited monocyte and macrophage chemotaxis, migration, and CCR5 expression, while CCL5 rescued this phenotype. Therefore, PTBP2 induced chemotaxis of tumour-associated monocytes and macrophages through the CCL5/CCR5 axis within the TME [20].

Moreover, the conditional medium obtained from coculturing PTBP2-overexpressing NB cells and monocytes was collected and subsequently added to naïve NB cells. This treatment led to reduced proliferation and migration of NB cells, outlining the impact of secreted molecules into this simulated TME [20].

**Figure 4:**
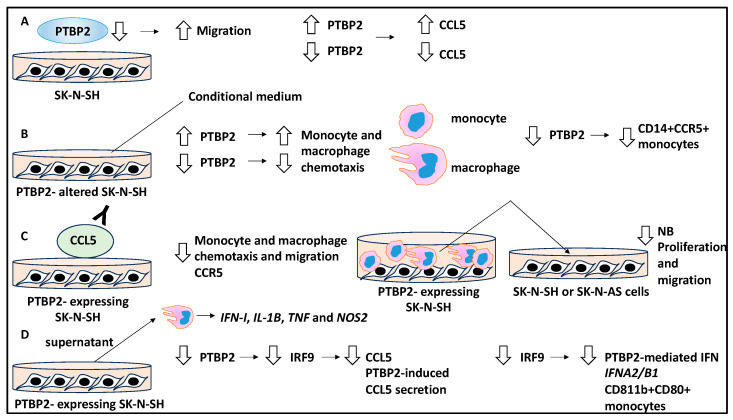
The role of differentially spliced RNAs in NB TME dynamics. (**A**) PTBP2 was detected in the SK-N-SH cell line, and the knockdown of this gene increased NB cell migration. The supernatant of SK-N-SH cells with altered PTBP2 expression showed modified levels of CCL5 cytokine in the TME (culture medium). Accordingly, overexpression or suppression of PTBP2 led to CCL5 up- and downregulation, respectively. (**B**) The conditional medium of PTBP2-altered SK-N-SH cells was collected and used to treat monocytes and macrophages, and this altered the chemotactic activity of these cells. For example, the expression and knockdown of PTBP2 led to increased and decreased macrophage and monocyte chemotaxis, respectively. Moreover, the knockdown of PTBP2 decreased CD14+ CCR5+ monocyte cell presence. (**C**) The blockage of CCL5 using an antibody in PTBP2-expressing SK-N-SH cells led to reduced monocyte and macrophage chemotaxis, migration, and CCR5 levels within the TME. Further, the conditional medium obtained from the coculture of PTBP2-altered NB cells and monocytes was used to induce naïve NB cells (for example, SK-N-SH or SK-N-AS cells), and this inhibited the proliferation and migration of NB cells. (**D**) Macrophages treated with conditional media from SK-N-SH-PTBP2-expressing cells were processed for RNA sequencing, and it was shown that *IFN-I, IL-1B, TNF,* and *NOS2* were differentially expressed. PTBP2 knockdown decreased IRF9 levels, while the knockdown of IRF9 led to the downregulation of CCL5 and PTBP2-mediated CCL5 secretion. Also, low levels of IRF9 decreased *IFNA2/B1* and CD11b+CD80+ monocytes.

Macrophages exposed to the conditional media from PTBP2-overexpressing NB cells were studied with RNA sequencing, and it was shown that *IFN-I*, *IL-1B*, *TNF*, and *NOS2* were differentially expressed by macrophages, suggesting the adoption of inflammatory phenotypes by these cells to in turn impact the TME [20]. Mechanistically, PTBP2 regulated the alternative splicing of interferon regulatory factor 9 (IRF9) since it could bind to the exon 6-7-8 regions of IRF9. Consistently, PTBP2 knockdown decreased IRF9 levels [20]. Also, the knockdown of IRF9 led to the downregulation of CCL5 and PTBP2-mediated CCL5 secretion, while these lower levels of IRF9 decreased IFNA2/B1 levels and CD11b+CD80+ monocytes, along with the PTBP2-mediated modulation of these factors and cells. In conclusion, PTBP2-mediated IRF9 splicing, which, per se, regulated CCL5 and *IFNA/B* secretion linked to monocyte chemotaxis in the TME and IFN-1 response [20]. Overall, a complex interaction between NB tumour cells, namely, a cascade of regulatory and secreted factors, chemokines, and cytokines downstream of the tumour suppressor PTBP2, affected monocytes and macrophages, and this could lead to the limiting of NB growth within the TME (Figure 4).

A similar study investigated the contribution of beta-1,3-galactosyltransferase (B3GALT4) to the NB TME. In this study, patient data deposited in publicly available databases and clinical NB samples were collected, and the expression of GD2 and B3GALT4 proteins was established [40]. Accordingly, GD2 was highly expressed in NB patients with poor prognosis, while the opposite trend was observed for B3GALT4 (the downregulation of this gene was linked to poor prognosis in NB patients). In addition, GD2 expression was regulated by B3GALT4 (a strong negative correlation was observed between these two genes). The silencing and upregulation of B3GALT4 led to increased and decreased tumour proliferation and migration in vitro of 9464D cells, respectively. The upregulation of B3GALT4 in vivo (nude mouse xenografted with B3GALT4-expressing 9464D cells) led to tumour regression and the recruitment of CD8+ T cells. Notably, the recruitment of CD8+ T cells was mediated through chemokines such as CXCL9 and CXCL10. The upregulation of B3GALT4 acted by targeting CXCL9 and CXCL10 and promoting their secretion to recruit CD8+ T cells via GD2 and c-Met/AKT/mTOR/IRF-1 signalling pathways [40]. In conclusion, B3GALT4 could modulate the recruitment of CD8+ cells to the TME through a myriad of chemokines and therefore might be a viable target for NB therapy. Overall, these two studies delineate the role of differential expression and various proteins in modulating the TME via regulating cytokines and chemokines.

### 2.5. The Role of RNA-Binding Proteins in the TME That Influence NB Metastasis

IGF2BP1, an RNA-binding protein and a potential proto-oncogene linked to cellular adhesion, is encoded by the 17q21-ter locus that is frequently detected in NB patients. Specifically, the gain on the 17q21-ter locus is linked to reduced progression-free survival, and, irrespective of MYCN status, is seen in almost 50% of NB cases [21]. Given the link between IGF2BP1 levels in NB patients with unfavourable prognoses, a study attempted to understand the contribution of this protein to NB metastasis using immunocompetent mouse models and to establish the role of EVs within the TME [21,24,41].

The authors developed a highly metastatic NB cell line by transplanting 9464D cells into C57Bl6 mice and harvested liver metastasis in one mouse, thereby establishing a cell line (M1). This cell line showed a penetrance of metastasis of 100% in the livers of their recipients; despite this, the in vitro profile of this cell line did not match that of the in vivo model and only displayed low proliferation and migration potential. This suggested that perhaps secreted EVs and their associated factors and proteins may have an impact on in vivo metastasis by remodelling the TME and affecting resident stromal cells. By that, EVs secreted by M1 cells administered to mice for three weeks, increased fibronectin expression and CD45+ cell infiltration in the liver, thereby contributing to the premetastatic niche (PMN) that is defined as a permissive metastatic environment [21]. This suggests that the EVs remodelled the TME to enhance and promote NB cell homing and metastasis.

The authors then linked IGF2BP1 with the M1 cell line by downregulating this protein, which resulted in reduced metastatic potential following xenografting of the mouse model with these cells and increased the survival of these mice (but did not affect tumour growth). These observations also applied to the Neuro2A cell line, in which the overexpression of IGF2BP1 by these cells increased their metastatic potential and reduced survival in vivo [21]. This suggested that IGF2BP1 acted as an oncogene to promote metastasis.

The authors then determined the role of EVs, their link to IGF2BP1, and whether these could impact metastasis. EVs of IGF2BP1 overexpressing or knockdown cells were isolated and characterised by nanoparticle tracking analysis. These EVs were within 100–200 nm and fell within the small range of EVs [21]. Subsequently, the authors treated mice with EVs derived from nontargeting M1 and IGF2BP1-knockdown M1, and it was shown that the former enhanced metastasis to the liver, while the latter did not. In addition, the nontargeting M1 cell EVs increased fibronectin and CD45+ cells (components of the PMN). Hence, IGF2BP1 upregulated fibronectin and CD45+ cell infiltration, suggesting the role of IGF2BP1 in EV-enhanced metastasis and preparing the metastasis niche for the easier homing of NB tumour cells [21].

**Figure 5:**
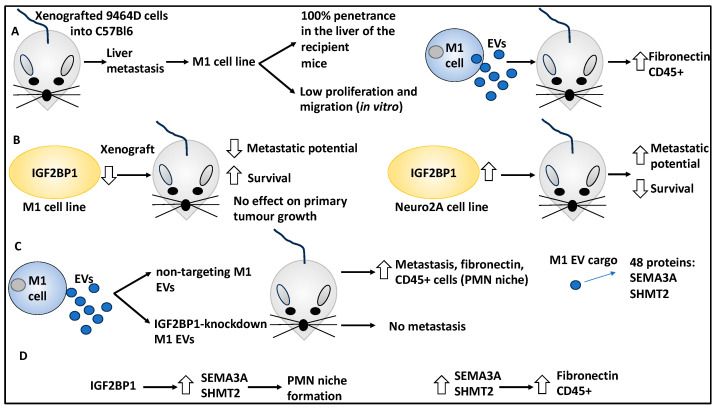
The role of IGF2BP1 in NB–immune cell–TME interplay. (**A**) The authors initially developed highly metastatic NB cells termed M1 by transplanting the 9464D cell line to C57Bl6 mice, then collecting and culturing the liver metastasis tumours. This cell line showed 100% liver metastasis penetrance once transplanted to new mice, although it showed low proliferation and migration rates in vitro. Subsequently, M1 cell line EVs were studied, and the EVs secreted by the M1 cell line were administered to mice for three weeks. These EVs increased liver metastasis by contributing to PMN due to CD45+ cell infiltration and fibronectin expression and therefore influenced the PMN. (**B**) The knockdown of IGF2BP1 followed by xenografting to the mouse model led to reduced metastatic potential and increased survival (tumour size was not affected). Overexpressing IGF2BP1 in Neuro2A cells followed by xenografting led to increased metastatic potential and decreased survival. (**C**) IGFBP1 was knocked down in the M1 cell line, the resulting EVs were compared to EVs from nontargeting controls, and it was shown that the former led to no metastasis while the latter led to metastasis and increased fibronectin and CD45+ cells. The protein cargo in the M1 EVs included SEMA3A and SHMT2 proteins. (**D**) IGF2BP1 increased the levels of SEMA3A and SHMT2 in EVs, and this promoted PMN formation. SEMA3A and SHMT2 led to increased CD45 and fibronectin in mouse model livers.

These EVs were also studied for protein cargo, and over 48 proteins were identified to be differentially presented between EVs derived from nontargeting M1 and IGF2BP1-knockdown M1 cells. Two such proteins were semaphorin 3A (SEMA3A) and serine hydroxymethyltransferase 2 (SHMT2) [21]. From a mechanistic viewpoint, IGF2BP1 increased the levels of SEMA3A and SHMT2 in EVs, and these proteins, per se, promoted PMN formation. Specifically, EVs derived from SEMA3A and SHMT2-overexpressing cells phenocopied IGF2BP1 overexpression and led to increased CD45 and fibronectin in mouse model livers [21]. In conclusion, the study relayed the function of IGFBP1 and the proteins within the EVs, including SHMT2 and SEMA3A, in influencing NB metastasis and PMN formation (Figure 5). From a TME viewpoint, the authors elegantly showed that various proteins and EVs can prime the secondary TMEs to facilitate metastasis and homing.

### 2.6. The Contribution of EVs to NB PMN and Metastasis

A study attempted to simulate the kinetics of the secondary TME, the PMN in NB, and the contribution of EVs to this dynamic [42,43].

Initially, TurboGFP NB cells were engineered by linking a domain of Lyn tyrosine kinase and GFP to an internal phospholipid membrane. GFP-expressing NB cells (XPack-GFP SK-N-SH NB cells) were imaged, and it was shown that the intracellular vesicles and plasma membrane expressed GFP. In addition, using ultracentrifugation and Western blots, tumour extracellular vesicles (TEVs) released by these cells were characterised, and it was shown that these TEVs were within the exosome size range (mean size of 94.7nm in diameter) and expressed GFP [43]. These TEVs expressed exosomal markers such as tetraspanin, ALIX, CD9, CD81, and CD63. The authors found microRNAs (e.g., *miR-1246*) in the RNA species isolated from these TEVs (using nanostring technology), and this was then used as proof of EV delivery. In addition, the coculture of mouse macrophages (RAW 264.7) with XPack-GFP SK-N-SH cells revealed the transfer of vesicular GFP to the macrophages, assessed using flow cytometry for human CD298 and mouse macrophage F4/80 markers (i.e., CD298−/F4/80+ cells) [43]. In addition, *miR-1246* was also detected in RAW264.7 cells [43]. This showed the interplay between the NB tumour cells and macrophages within this simulated TME.

Further, the authors attempted to understand the contribution of these TEVs to the PMN in vivo by monitoring the release of these TEVs in mice xenografted with NB tumour cells. Initially, these TEVs were isolated from the plasma samples of xenografted mice and studied with ExoView and Nanosight technologies to establish the characteristics of these vesicles. For example, it was shown that the mean size was 92.9 nm (consistent with EV sizes) and also expressed tetraspanins CD9, CD81, and CD63 [43]. Also, having xenografted the liver metastasis model with SK-N-BE(2)^Liv^ XPack-GFP NB cells, the presence of *miR-1246*-containing EVs in the plasma of these mice was detected on day 28 [43]. GFP+ TEV-capturing F4/80+ macrophages were tracked in the livers of these mice using time-lapse fluorescent microscopy at day 14, although tumour cells did not appear in this location until day 28, suggesting priming and preparation functions by the macrophage to allow easier homing of the tumour cells within the new TME [43]. In addition, the progressive homing of the NB tumour cells was established by flow cytometry; accordingly, F4/80+ macrophages were detected in the liver before the detection of CD298+ GFP+ tumour cells. Interestingly, other resident cells in the mouse liver, including hepatic stellate cells and hepatocytes, presented with GFP, indicating that other cells could capture TEVs. Overall, the capture of TEVs by the stromal cells located in the environment preceded the metastatic homing of the tumour cells, suggesting an apparent priming of the TME/PMN by these cells [43].

**Figure 6:**
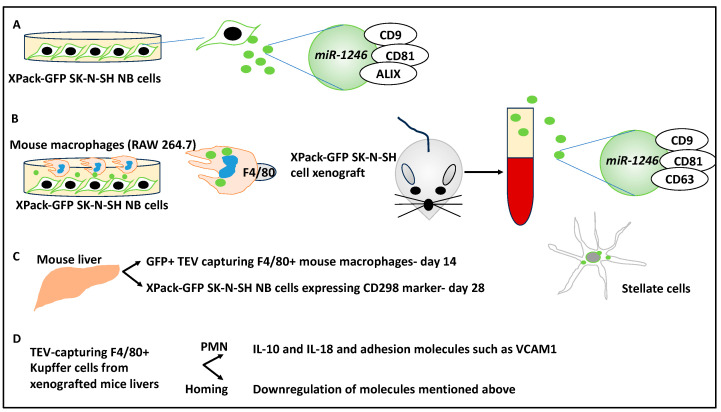
TEVs secreted from NB affect the PMN. (**A**) GFP-expressing NB cells (XPack-GFP SK-N-SH NB cells) were imaged, and it was shown that the intracellular vesicles and plasma membrane expressed GFP. These cells secreted EVs that expressed ALIX, CD9, CD81, and CD63 and also encapsulated *miR-1246*. (**B**) The coculture of mouse RAW 264.7 macrophages with XPack-GFP SK-N-SH cells showed the transfer of vesicular GFP to these macrophages (expressing F4/80 markers). XPack-GFP SK-N-SH was xenografted to nude mice, and the plasma of these mice was studied and TEVs were isolated. These TEVs expressed CD9, CD81, and CD63 and contained *miR-1246.* (**C**) Following xenografting of SK-N-SH^Liv^ XPack-GFP cells to the liver metastasis mouse model, the liver was monitored by microscopy, and GFP+ TEV-capturing F4/80+ macrophages were detected at day 14; yet, NB tumour cells were only detected on day 28. Accordingly, F4/80+ cells appeared in the liver before CD298+GFP+ tumour cells. Stellate cells of the liver also presented with GFP (and *miR-1246*), suggesting the capture of TEVs by these stromal cells. (**D**) TEV-capturing F4/80+ Kupffer cells were then isolated from xenografted mouse livers and divided into PMN and homing stages. In the former, cytokines including IL-10 and IL-18 and adhesion molecules such as VCAM1 were upregulated, while these genes receded after homing, suggesting an evolving process of colonisation.

Furthermore, the capture of TEVs was linked to specific organs since GFP-expressing tumour cells and F4/80+ GFP+ mouse macrophages were only detected in the liver but not the lungs, kidney, or brain (although low levels were also seen in the bone marrow). In addition, it was shown that TEV capture at the metastasis sites was associated with *miR-1246* capture by liver macrophages and stellate cells [43]. Overall, this suggested that organ-specific and selective TEV capture occurred. TEV-capturing F4/80+ Kupffer cells were then isolated from xenografted mouse livers and split into PMN and homing stages. In the PMN stage, macrophage markers (CD86, CD4, and CD86), cytokines including IL-10 and IL-18, and adhesion molecules such as VCAM1 were upregulated, while these genes receded after colonisation was accomplished [43]. In conclusion, TEV-capturing cells contributed to the processes of PMN priming and induced changes to the inflammatory repertoire, which facilitated the homing of NB tumour cells. Once colonisation was completed, the cytokine and adhesion molecules were downregulated, and therefore, the PMN evolved into a metastatic niche (Figure 6).

Other studies have also shown that miRNAs can be encapsulated into EVs within the TME. For example, *miR-574-5p* may be sorted into EVs and secreted from NB tumour cells, and in a paracrine fashion, they could, in turn, alter the TME. For example, these *miR-574-5p*-encapsulated EVs increase α-SMA in the fibroblasts and impact their differentiation and positive contribution to NB progression [44].

### 2.7. Immune Cell Infiltration to the TME May Contribute to NB Relapse

Many high-risk NB tumours will initially respond to treatment, and most NB patients will display remission; however, relapse may still occur due to the dominance of treatment-resistant clones [11,12].

A study aimed to understand the contribution of immune processes and cells to MYCN-amplified NB relapse. Accordingly, 46 samples from 22 NB patients with MYCN amplification before and after treatment were analysed for global gene expression using RNA sequencing and the enrichment of gene ontology terms [45]. To that effect, a subset of upregulated biological processes was linked to post-treatment MYCN amplification and included immune-related terms such as IL-2-STAT5 signalling, IFN-γ-response, macrophages, and T-cell recruitment, suggesting an elevated inflammatory profile that is triggered by the components of the TME post-treatment to reduce its efficacy [45].

Furthermore, MYCN-amplified NB tumours post-treatment were stained with chromogranin A and CD45, which showed the presence of immune cells. In addition, the GeoMx high-resolution proteomics platforms allowed for the spatial resolution and digital quantification of proteins and revealed the infiltration of monocytes and macrophages with the markers of CD14, CD11c, CD68, and HLA-DR in post-treatment MYCN-amplified samples. Interestingly, there was an upregulation of proteins linked to immunosuppression, including CD163, Tim-3, and B7-H3 [45], suggesting that the protumoural infiltration of immune cells into the TME and a repertoire of immunosuppressive factors post-treatment could induce immunosuppression.

The mechanism by which macrophages infiltrated the TME was through the increase in colony-stimulating factor (CSF1) and chemokine ligand 2 (CCL2) (both involved in macrophage recruitment) in post-treatment MYCN-amplified tumours [45,46]. Consistently, post-COJEC NB samples also revealed enhanced gene expression levels of CCL2 and a mesenchymal phenotype, consistent with the expansion of CCL2+ NB tumour cells [45].

Unlike the pretreatment MYCN-amplified tumours, the post-treatment MYCN-amplified tumours displayed dramatic alterations in the genetic landscape, including changes to the number of copy number alterations, which correlated with macrophage infiltration, suggesting tumour evolution within the altering TME [45].

**Figure 7:**
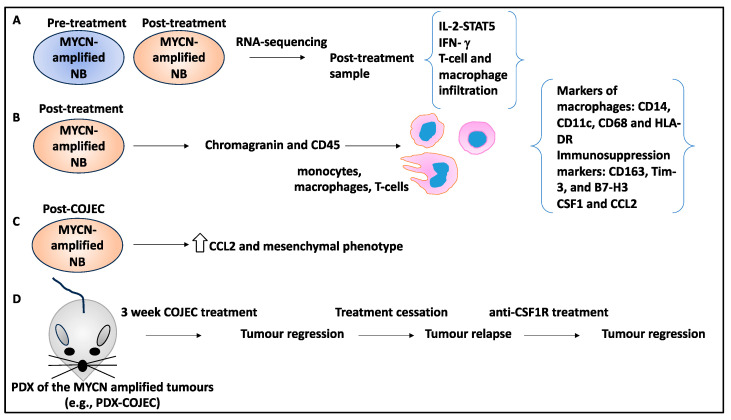
The contribution of immune cells and responses to relapse in MYCN-amplified NB tumour samples. (**A**) Pre- and post-treatment MYCN-amplified NB patient tumour samples were processed by RNA sequencing, and the presence of T cells and macrophages and upregulation of biological pathways such as IL-2-STAT5 signalling and IFN-γ response were observed with the post-treatment NB patient tumour samples (TME). (**B**) The post-treatment MYCN-amplified NB samples showed the presence of immune cells following staining for CD45 and chromogranin markers. The GeoMx high-resolution proteomics platform showed the infiltration of monocytes and macrophages expressing CD14, CD11c, CD68, and HLA-DR markers. In addition, immunosuppression markers including CD163, Tim-3, and B7-H3 were upregulated. Macrophage infiltration was facilitated by the increase in CSF1 and CCL2 in post-treatment MYCN-amplified tumours. (**C**) Post-COJEC NB samples also revealed enhanced gene expression levels of CCL2 and a mesenchymal phenotype. (**D**) Cells from an MYCN-amplified PDX (PDX#3) were xenografted, and the mice received COJEC for three weeks. Tumour relapse was allowed to occur by ceasing treatment for three weeks, after which anti-CSF1R treatment was initiated, which led to reduced tumour relapse.

Given the importance of CSF1 in macrophage recruitment to the TME, MYCN-amplified PDX-COJEC was xenografted to a nude mouse model to test the effect of anti-CSF1R treatment on inhibiting macrophage recruitment to NB tumours [45]. Accordingly, dissociated organoids from a previous PDX (PDX#3) were xenografted, and the mice received COJEC for three weeks. Tumour relapse was allowed to occur by ceasing treatment for three weeks, after which anti-CSF1R treatment was initiated, which led to reduced tumour relapse and growth [45]. In conclusion, this study revealed that the regrowth of MYCN-amplified tumours post-COJEC is influenced by the infiltration of macrophages to the TME, and inhibiting macrophage recruitment can be a viable follow-up treatment option for post-treatment relapse in high-risk NB (Figure 7).

### 2.8. Infiltration of Immune Cells to Spleens and TME Post-Treatment Linked to NB Treatment

The interaction of T-cell programmed cell death protein 1 (PD-1) with its tumour cell ligand can lead to decreases in antitumour response by these T cells, hence the use of anti-PD-1/PD-L1 antibodies for improving treatment response [47]. A study attempted to understand the link between the type and density of tumour-infiltrating lymphocytes in the spleens and TME following checkpoint inhibition by the anti-PD-1/PD-L1 antibody coadministration in NB [48]. The authors showed that the coadministration of anti-PD-1/PD-L1 antibodies did not affect tumour growth in nude mice (lacking T cells; BALB/c slc-nu/nu) xenografted with Neuro2A NB cells, suggesting that T cells are important for the efficacy of this treatment [48]. The authors then used an inbred A/J mouse model, which had T-cell function and was genetically linked to Neuro2A cells, while tumour growth and T-cell infiltration in spleens were assessed using flow cytometry following the administration of five doses of the combination of anti-PD-1/PD-L1 antibodies. This treatment suppressed tumour growth in this model, since the median tumour weight at day 16 was lower in the treatment versus control groups. The treatment response (following anti-PD-1/PD-L1 antibody coadministration) to that effect was also categorised into three groups of marked, mild, and no effect based on tumour weight of <300 mg, 300–600 mg, and 600 mg, respectively, and these were compared to an isotype control group [48]. From the viewpoint of the effect of treatment on the lymphocytes in the mouse spleen, the study revealed that regulatory T cells (CD4+CD25+FoxP3+) were not significantly altered in treatment versus control groups (i.e., treatment naïve and isotype control) [48]. This observation also applied to CD69-expressing CD4+ lymphocytes (CD69 is a marker of lymphocyte activation) since these cells were not affected in the spleens of treatment and control mice (although there was a trend for higher CD4+ CD69+ cells in the affective treatment group with tumours smaller than 300 mg compared to mild or no-effect groups). Despite this, the percentage of CD69-expressing CD8+ lymphocytes in the spleens of the affective treatment group with tumours smaller than 300 mg was statistically higher than the mild and no-effect groups (>300 mg tumour size) and isotype control [48]. Finally, the authors revealed that activated CD8+CD69+ lymphocyte infiltration into the spleens correlated inversely with the tumour weight [48]. In addition, the tumour-infiltrating CD8+CD69+ expressing cells were negative for CD49b markers, suggesting these were not NK cells but rather activated lymphocytes [48]. It also followed that the infiltration of CD8+CD69+ cells into the TME in the effective groups was also significant compared to the other study groups.

**Figure 8:**
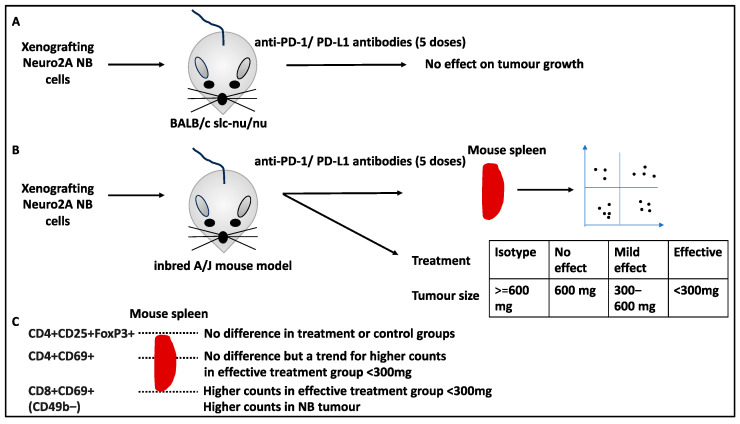
The infiltration of immune cells to the spleen and the TME following anti-PD-1/PD-L antibody coadministration. (**A**) The xenografting of Neuro2A NB cells to nude mice lacking T cells, e.g., BALB/c slc-nu/nu mice, followed by 5 doses of treatment (anti-PD-1/PD-L1 antibodies) did not affect tumour growth. (**B**) The xenografting of Neuro2A NB cells into an inbred A/J mouse model and the infiltration of lymphocytes to the spleen were monitored using flow cytometry, while tumour sizes were measured. Concerning tumour size post-treatment, four groups were recognised. Isotype control (and treatment naïve controls) did not affect tumour growth, while treatment groups were classified as no-effect, mild, and effective treatment groups ranging in tumour size post-treatment (i.e., <300 mg, 300–600 mg, and 600 mg, respectively). (**C**) Lymphocyte infiltration to the spleen was monitored, and the study revealed that regulatory T cells (CD4+CD25+FoxP3+) were not altered across study groups. This applied to CD4+CD69+ cells (although a trend for higher counts was observed in the effective treatment group). The CD8+CD69+ (and CD49b−) cells, however, were statistically higher in the spleens of the effective treatment group, and this applied to the infiltration of these cells to the NB tumour as well.

In conclusion, this study outlined the contribution of lymphocytes as important TME cells to antitumour responses implemented by blocking PD-1/PD-L1. This also suggested that encouraging the infiltration of activated CD8+ lymphocytes to the TME may improve treatment response, unravelling an important aspect of the NB treatment [48] (Figure 8).

### 2.9. Small Molecule Inhibitors Could Stimulate an Immune Response in the TME

In high-risk NB patients, the GD2 antibody treatment induced antibody-dependent cell-mediated cytotoxicity (ADCC), and the success of this treatment was linked to the presence of immune cells, including NK cells [49]. CD38 is a marker of NK cells within the TME and possesses hydrolase activity, and CD38 overexpression may be linked to immunosuppression; hence, the authors of the study aimed to block this molecule using a small molecule inhibitor to overcome immunosuppression in the TME that may in turn impact NB treatment [50].

Initially, the authors tested CD38, CD203a, and CD73 expression levels in 786 NB patient samples, and a strong positive correlation between these markers was detected (all three markers are indicated in alternative adenosine pathways linked to immunosuppression). The authors developed and tested multiple small molecule inhibitors targeting CD38 and established their respective IC50s. Further, the CD38 hydrolase inhibitor 2 (referred to as 2) was tested on human peripheral blood NK cells, and this treatment led to the expansion of these cells [50]. Furthermore, the authors detected a variation in IFN-γ levels in NK cells (obtained from various human donors) when cultured in the presence of compounds **2** and **14** (compound **14** was a known CD38 inhibitor used as a positive control). Despite the variation, all donors responded in a concentration-dependent manner to compounds **2** and **14**, since increasing the compound dose increased IFN-γ levels [50]. The authors also tested the effect of inhibiting CD38 hydrolases on the activity of NK cells within this simulated TME over a long period (20 days of culture with 3-day medium exchange intervals), and NK cells treated with compounds **2** and **14** expressed increased IFN-γ levels, suggesting that inhibiting CD38 hydrolases enhanced NK cell viability and function [50]. Accordingly, since the TME downregulated NK cell activation over time, inhibition of the activity of CD38 hydrolase therefore enhanced NK cell activation, and this is therapeutically relevant.

Due to the initial success of inhibiting CD38 hydrolase in enhancing NK function and activity, the authors tested an immunotherapy regimen consisting of an anti-GD2 antibody domain (ch14.18) linked to IL-2. Ch14.18-IL2 in combination with an inhibitor of CD38 hydrolase to assess its antitumour effects. To that effect, SH-SY5Y-GFP cells were cultured and then exposed to NK cells with another fluorescent tracker in the presence of 50 ng/mg of ch14.18-IL-2 with compounds **2** or **14**. After 90 min of coculture, fluorescent imaging revealed that there was a significant decrease in the SH-SY5Y-GFP cell count (e.g., 1 mM of compounds **2** and **50** ng/mL of ch14.18-IL-2 caused a circa 14% decrease in the fluorescent area of NB cells compared to controls). This did not apply to 1 mM of compound **14** [50]. This suggested that this treatment increased the cytotoxic activity of NK cells.

**Figure 9:**
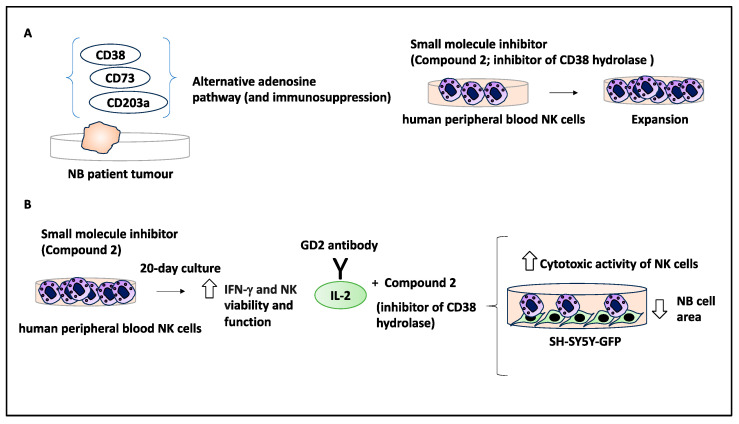
The combination of small molecule inhibitors of CD38 hydrolase and GD2 antibodies in priming NK cells to overcome immunosuppression in the TME and reduce NB tumour growth. (**A**) CD38, CD203a, and CD73 were expressed in 786 NB patient samples, and these markers were indicated in an alternative adenosine pathway that is linked to immunosuppression. In addition, small molecule inhibitors of CD38 hydrolase (for example, compound **2**) were tested (since CD38 possesses hydrolase activity). Treating human peripheral blood NK cells with compound **2** led to the expansion of these cells. (**B**) A 20-day culture of human peripheral blood NK cells with compound **2** also led to increased levels of IFN-γ and NK cell viability and function. In addition, the authors tested the combination of anti-GD2 antibodies linked to IL-2 combined with compound **2**; the coculture of NK cells and SH-SY5Y-GFP cells in the presence of this combination treatment led to reduced NB cells and increased cytotoxic activity of NK cells.

In conclusion, the authors developed and tested compounds and showed that compound **2** (IC50 = 1.9 uM) was an inhibitor of CD38 that induced IFN-γ and thereby promoted the proliferation of NK cells in the TME. The coculture of NB/NK cells in the presence of compound **2** and **ch14**.18-IL-2 reduced NB cell count, and this combination may be used to modulate the NK cell activity as a key component of the TME to limit tumour growth [50] (Figure 9). Overall, modulating NK cells to overcome immunosuppression and limit NB growth may indeed be a useful treatment strategy.

### 2.10. Targeting Soluble Ligands in the TME to Overcome Immunosuppression

NB tumours may secrete proteins such as NKG2D ligands (NKG2L), which may allow for immune evasion and tumour cell escape from immunological surveillance within the TME. As evidence of this, NKG2D is regarded as a costimulator of T-cell receptors, while NKG2DL, on the other hand, comprises MICA/B and ULBP-1-6 proteins that are expressed by malignant cells to impair NK and T cell-mediated responses by triggering the internalisation of NKG2D receptors in NK and T cells [51]. This leads to a more immunosuppressive and tumour-friendly TME.

Given this background, a study aimed to understand the role of NKG2DL, MICA/B, and ULBP1-6 proteins in immunosuppression in the TME. Accordingly, the authors measured the levels of soluble NKG2DL, MICA/B, and ULBP1-6 binding proteins in 35 NB patients and 10 healthy volunteer serum samples [28]. Accordingly, the serum levels of MICA and ULBP2 were much higher in NB patients than in healthy volunteers, indicating these ligands may have been produced to trigger immune escape in these tumours. In addition, MICA/B and ULBP1-3 were detected in SH-SY5Y and SK-N-BE2 cell line supernatant, and immune cells were exposed to this supernatant that contained NKG2DL [28]. In turn, NKG2DL was cleaved by ADAM10 and ADAM17, since using inhibitors for these proteins reduced the shedding of the mentioned NKG2DLs. Further, it was also shown that in the serum of postoperative NB patients, the levels of soluble MICA and ULBP2 were reduced compared to preoperative patients, suggesting that serum NKG2DL levels correlated with an unfavourable patient prognosis [28].

From the viewpoint of the TME cells, the effect of NKGD2 on lymphocytes, including NK cells and CD8+ T cells, was evaluated. Accordingly, the expression of NKG2D receptors of these cells having been exposed to the NB tumour supernatant for 12 h was monitored by FACS [28]. It was shown that CD8+ T cells downregulated NKG2D receptors following exposure to the NB cell supernatant. In addition, the higher the concentration of the supernatant, the lower the levels of NKG2D. From a functional viewpoint, NKG2D downregulation of CD8+ T-cells by exposure to NB cell supernatants for 12 h was also accompanied by reduced CD8+ T-cell proliferation and IFN-γ production by these cells (due to NKG2DL downregulation of CD8+ T-cell function). Furthermore, the percentage of CD8+ T cells that expressed IFN-γ, Ki-67, and CD107 was reduced, and this was more profound in cells treated with higher concentrations of NB cell supernatant [28], suggesting the role of TME proteins and factors in priming immune cells.

**Figure 10:**
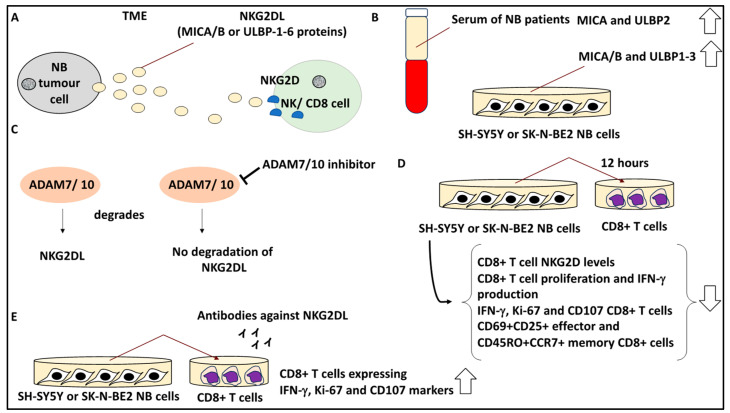
Targeting soluble NKG2DL can reverse immunosuppression. (**A**) NB tumours express a myriad of NKG2D ligands (NKG2DLs) including MICA/B and ULBP-1-6 proteins, and these lead to the internalisation of NKG2D receptors and reduced immune response by NK and CD8+ T cells. (**B**) NKG2DLs were measured in the serum of NB patients, and it was shown that MICA and ULBP2 were upregulated compared to healthy control patient sera. Also, the supernatant of SH-SY5Y and SK-N-BE2 cells showed high levels of MICA/B and ULBP1-3. (**C**) NKG2DLs were cleaved by ADAM7 and 10, and this could be blocked by using ADAM7/10 inhibitors. (**D**) The treatment of CD8+ T cells with the supernatant of NB cells led to reduced CD8+ cell NKG2D expression levels, in addition to the reduction in CD8+ T-cell proliferation and IFN-γ production. The percentage of IFN-γ, Ki-67, and CD107-producing CD8+ T cells was lowered. In addition, CD69+CD25+ effector and CD45RO+CCR7+ memory CD8+ cells were reduced. (**E**) The inhibition of NKG2DL using a combination of ULBP1/2/3 and MICA/B antibodies led to increased percentages of CD8+ T cells expressing IFN-γ, Ki-67, and CD107 markers.

Interestingly, the authors tested whether both effectors and memory CD8+T cells may be affected by NKG2DL, and it was shown that the percentage of CD69+CD25+ effector and CD45RO+CCR7+ memory CD8+ T cells was reduced after exposure to NB cell culture supernatants. Overall, this suggested that both effector and memory formation were impaired by NKG2DL [28]. Finally, the blockage of NKG2DL using a combination of antibodies against NKG2DL, including anti-ULBP1/2/3 and MICA/B, led to increased percentages of CD8+ T cells expressing IFN-γ, Ki-67, and CD107 markers [28]. In conclusion, blocking sNKG2DL using specific antibodies to this soluble ligand can be an important strategy to enhance T-cell function within the TME and thereby improve antitumour immunotherapy (Figure 10).

In addition, overcoming immunosuppression can be accomplished by physically linking the human 14.18 antibody to IL-15 or IL-2 (i.e., immunocytokine) to induce antibody-mediated cytotoxicity, with NK cells serving as effector cells [52]. As evidence of this, hu14.18-1l-15 and hu14.18-IL2 activated NK cells, and as a result, led to cytotoxic effects on GD2-expressing NB cells, and both immunokines maintained the immunolytic effects of NK cells against NB tumour cells [52]. This was also tested in orthotopic xenograft nude mice with NK cells as effector cells, and it was shown that the immunocytokines combined with chemotherapy could induce tumour regression. Hu14.18-1l-15 outperformed hu14.18-IL2 when the study subject was a syngenetic immunocompetent mouse, suggesting immune cells other than NK cells may also contribute to the therapeutic function. These tumours contained CD8+ T cells and M1 macrophages and limited Tregs and MDSCs in the TME, suggesting the potential reversal of immunosuppression. In conclusion, it is possible to attempt to overcome the immunosuppressive TME using these fused cytokines as adjunct molecules to monoclonal antibodies, and NK cells may play a key role in antitumour responses in the TME [52].

### 2.11. Other Aspects of NB Interaction with the TME (CAR T Cells and Hypoxia)

Chimeric antigen receptor (CAR) T cells targeting GPC2 were tested to reduce minimal residual disease in NB patients. Accordingly, GPC2-CAR constructs were engineered, and their activity was tested in NOD-SCID mice orthotopically xenografted with NB cell lines [53]. As evidence of this, GPC2 is a foetal antigen involved in development and is lowly expressed in NB, making it a relatively unique target. It was shown in vivo that the CAR vector containing the single-chain variable fragment (CT3), CD28 hinge and transmembrane and 4-1BB domain (CT3.28H.BBζ) showed the most optimum antitumour effects, and the tumour regressed after 4 weeks of CAR T-cell infusion [53]. In addition, GPC2-CAR T cells were subjected to single-cell RNA sequencing. Accordingly, the T-cell injection product of donor 2 before injection to mice comprised proliferating CD8+ and CD4+ T cells and CD8+ effector T cells (similar to donor 1, who both participated in this study). Also, after injection of these products into mice bearing IMR5 NB cells, on day 8, these tumours contained CD8 and CD4 effector T cells in the TME [53]. In addition, this upregulation of CD8+ T cells in donor 2 was also linked to the expression of several key genes encoding effector proteins, such as *ZNF683, HMGN2, GZMB,* and *GNLY*, by these CAR T cells. In conclusion, the finetuning of the CAR T-cell designs may lead to changes in the landscape of immune cells within the TME favouring immune activation and therefore may assist in the better targeting of NB tumour cells [53].

Hypoxia is one of the aspects of the TME, and a study aimed to investigate whether there was a link between SH-SY5Y cells treated with atorvastatin and hypoxia and the expression of the *HIF-1α* gene [54]. Given this background, the authors assessed different concentrations of atorvastatin in the presence and absence of hypoxia (induced by CoCl2) and the presence and absence of *HIF-1α* gene expression (manipulated by *HIF-1α* inhibitors). The study showed that atorvastatin had cytotoxic effects on NB cells, but under hypoxic conditions, an atorvastatin-induced increase in NB cell proliferation was observed [54]. Accordingly, 10 nM and 20 nM of atorvastatin combined with CoCl2 increased viability to 96.67% and 91.78% compared to the vehicle at 48 h post-treatment, respectively [54]. Since hypoxia may indeed occur in the TME, the link between atorvastatin as a drug that may induce cytotoxicity in NB but increase proliferation under hypoxic conditions is particularly interesting. In conclusion, this subsection highlighted the alterations that CAR T cells may induce in the TME that will impact NB treatment, while the opposing actions of a statin were reported when hypoxia was induced in the simulated TME.

Overall, in Section 2, the landscape of NB tumours from the viewpoint of immune and stromal cells and the repertoire of proteins, noncoding RNA, cytokines, chemokines, and vesicles that they could produce to bilaterally interact with and influence the ecosystem of the TME were dissected. It was also noted that the biophysical properties of the ECM could impact NB tumour cells. Multiple treatment options that targeted immunosuppression or the activation of immune cells and their infiltration into the TME were also comprehensively discussed. These interactions were also linked to NB biology, prognosis, treatment, relapse, and progression.

## 3. Discussion

**Figure 11:**
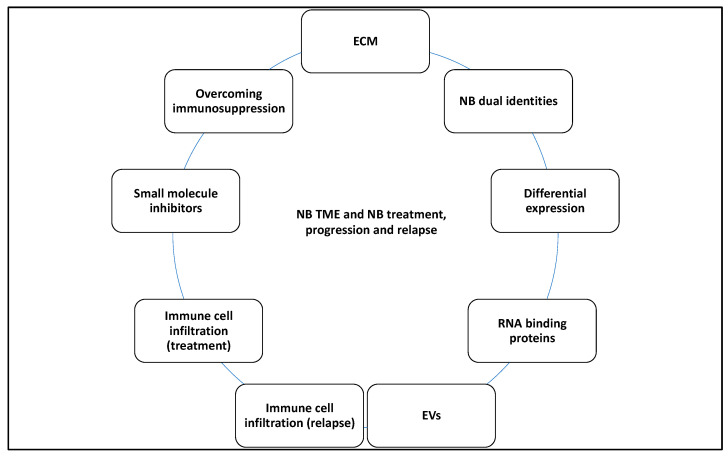
Review summary. The current study investigated the link between NB TME and NB biology, treatment, progression, and relapse with eight molecular structures, molecules, processes, or players, including the ECM, NB identities, differential expression of RNA, RNA-binding proteins, EVs, immune cell infiltration from a viewpoint of effect on relapse, cell infiltration from a viewpoint of effect on treatment, small molecule inhibitor-based treatment, and strategies to reverse immunosuppression.

This study attempted to understand the contribution of various factors, molecules, and mechanisms to the NB-TME dynamics that ultimately affected NB biology, treatment, prognosis, progression, and relapse (Figure 11).

Initially, the biophysical characteristics of the ECM as a major component of the TME were addressed. Tumour cells respond to physical cues, traction, and compression within the TME [33,34,35]. As discussed, the interaction between tumour cells and the ECM in the TME is bilateral, whereby tumour cells can adapt to the ECM stiffness and remodel it to a more favourable environment, while changes in the ECM can impact the genomic stability of tumour cells [55,56]. Similarly, a study on NB allowed the simulation and recapitulation of bone metastasis by using a 3D culture comprising collagen, glycosaminoglycans, and nanohydroxyapatite scaffolding, outlining the importance of the ECM composition as a key component of the TME in many aspects of the tumour biology, including metastasis [57].

NB contains fluid noradrenergic and mesenchymal epigenetic cell states [19]. Accordingly, the cell phenotypes for noradrenergic (e.g., DBH and PHOX2B expression) and mesenchymal/neural crest (COL1A1 expression) were obtained by the Van Groningen and Boeva studies [23,36,37,58]. Consistently, a single-cell RNA-sequencing study displayed substantial heterogeneity within the NB tumours and showed that neuroblasts transitioned between noradrenergic and mesenchymal states through a transitional neuroblast state, which the authors referred to as the transitional cells [23]. Single-cell transcriptomics, followed by principal component analyses, revealed strong inverse associations between noradrenergic and mesenchymal signatures and the transitional cells (perhaps reflecting the transition points between the two states) [23]. This transitional cell subpopulation expressed markers such as *MYCN*, *EZH2*, and *SOX11* and was associated with rapid proliferation and tumour metastasis. This gene expression profile was linked to an unfavourable patient prognosis compared to either noradrenergic or mesenchymal states. Finally, the NB tumours as a whole showed a trend for lower infiltration of T cells within the TME (e.g., CD8+ cells) [23], while macrophages with noninflammatory phenotype (M2) were abundant (assayed by probing for CD163), suggesting an overall immunosuppressive environment [23,30,31]. Overall, the NB tumour TME showed fewer CD8 T cells and a higher macrophage presence, linking NB with the TME, while NB identities and transitional states were linked to patient outcome [23].

The differential levels of PTBP2 and IRF9 induced chemotaxis of tumour-associated monocytes and macrophages through chemokines and thereby limited NB growth [20]. Similarly, B3GALT4 enhanced the recruitment of CD8+ T cells through other chemokines and the c-Met/AKT/mTOR/IRF1 signalling pathway [40]. Other studies have shown that differential expression due to alternative splicing can take place in *TrkA* variants (receptors for nerve growth factors indicated in nervous system development), including *TrkAIII*, which skips exons 6-7 in the TME. *TrkA* usually displayed tumour suppressor roles in NB and was linked to more favourable prognoses, while its splicing variant, *TrkAIII* on the other hand, was linked to unfavourable prognoses, metastatic disease, and disease relapse [59]. Overall, transcriptional variants and various proteins may facilitate oncogenic transformation within the TME backdrop.

The role of RNA-binding proteins such as IGF2BP1 in influencing the NB TME was discussed earlier, while patients with metastasis and poor prognosis upregulated this protein [21]. Interestingly, neoantigens may also be linked to patient prognoses and responses to treatment [60]. A study aimed to understand the prognostic role of the T-cell inflamed signature (dendritic cell and CD8+ cell gene expression profiles) and neoantigens in high-risk NB. The authors used the TARGET and GMKF databases to demarcate T cell-inflamed and non-T cell-inflamed group associations with MYCN amplification and patient survival, in addition to the link between neoantigen load and patient survival [60]. Patients with T cell-inflamed NB tumours displayed enhanced survival compared to their non-T cell-inflamed counterparts, irrespective of MYCN amplification status, suggesting the direct impact of the TME components on patient prognosis. Additionally, higher neoantigen loads were also linked to improved patient survival. Finally, the expression of *MYCN*, *SOX11*, and *ASCL1* was inversely correlated with T cell-inflated status. Overall, this study linked components of the TME, including proteins, neoantigens and immune cells, with prognosis in NB [60].

TEV-capturing cells in TME contributed to the PMN and induced changes to the inflammatory repertoire [43]. In addition, TEVs can alter the response to cancer treatment within the TME [61]. A study attempted to profile the function of TEVs in promoting resistance to dinutuximab (anti-GD2 monoclonal antibody) [61]. As evidence of this, 9464D cells were derived from a TH-MYCN mouse on a C57Bl6 background, while 9464D-GD2 (9464-D cell line expressing GD2) was used to derive the TEVs used in this study. C57Bl6 mice were xenografted with 9464D-GD2 cells and treated with dinutuximab and/or TEVs produced by and isolated from 9464D-GD2 cells. These TEVs reduced the efficacy of dinutuximab since a complete resistance to the antitumour effect of this drug was observed. Flow cytometry established that the dinutuximab and TEV combination skewed the TME towards containing a higher level of TAMs and fewer NK cells, suggesting the direct effect of this treatment on the TME immune cells [61]. Interestingly, the combination of tipifarnib (inhibitor of TEV secretion) sensitised NB cells in vivo to dinutuximab, inhibited tumour growth, and reversed the immunosuppressive effects asserted by these TEVs (e.g., TAMs were reduced). Overall, these studies highlighted the immunosuppressive effects of the infiltration of TAMs in the TME that can be overcome and the role of TEVs in shaping the PMN as a secondary TME site [61].

The link between immune cell infiltration and NB relapse was also reviewed [45]. In agreement with this, another study attempted to link immune cell infiltration to the TME with immune evasion and resistance to treatment [62]. Initially, MYCN-amplified NB9464 cells were xenografted to C57Bl6 mice, but only a rare infiltration of CD3+ or CD8+ T cells was observed, while macrophages were present. Furthermore, Syk, an immunosuppressive protein, skewed the macrophages towards immunosuppression (e.g., by the expression of *Mmp9*, *Arg*, and *Vegf*). The absence of Syk in mice (Syk^MC^-^KO^) xenografted with the NB9464 cell line led to tumour growth reduction, since the blockade of Syk remodelled the TME towards immune activation. In addition, the pharmacological blockade of Syk using R788, alone or in combination with PD-L1 antibody, led to tumour regression in the mouse model bearing tumours [62]. The combination of radiotherapy, R788, and anti-PD-L1 antibody substantially increased the survival of these mice. Overall, the study demonstrated that modulating Syk played a role in priming macrophages within the TME, while blocking Syk remodelled the TME towards an immune active state, outlining key features of NB–immune cell interactions within the TME [62].

The link between NB treatment (e.g., PDL-1 modulation) and the infiltration of immune cells was addressed in this review [48]. Consistently, using a mouse syngeneic model for NB, it was shown that SR59230A (an antagonist of β3-AR) led to reduced tumour growth and reduced levels of PD-L1 expression of the NB-bearing mice in the TME. Antagonism of β3-AR led to an increase in CD8+, dendritic, and NK cells and a reduction in T-reg cells and MDSCs in the TME, suggesting how inhibiting this receptor changed the TME ecosystem [7]. PD-L1 antibody and SR59230A treatment increased the production of granzyme B and perforin by PD1+CD8+ cells in the tumour mass, thereby stimulating immune activation in NB TME. Mechanistically, in NB tumour-bearing mice treated with β3-AR antagonists, tumour-infiltrating lymphocytes reduced IFN-γ secretion, and this also limited their ability to trigger PD-L1 expression by NB cells [7]. The study indicated the role of adrenergic receptors in influencing the TME by affecting tumour and immune cell interactions [7].

The use of anti-PD-L1 treatment for NB, including high-risk NB, and its links to the TME were discussed in this review [50], while other studies reported that an extraterminal domain bromodomain inhibitor (JQ1) and anti-PD-L1 combination treatment may be useful for treating high-risk NB [27]. Initially, using NB cell lines, the study showed that JQ1 inhibited HIF1α. Further, the TH-MYCN NB mouse model was screened by MRI and the relaxation rate R2* to measure hypoxia, and it was shown that JQ1 treatment reduced tumour volume and hypoxia [27]. From the viewpoint of blood vessel integrity, hypoxia microvascular networks are primitive, leaky, and fragile, with poor pericyte and basement membrane structure [27]. JQ1 treatment of these tumours led to improved blood vessel structure and integrity (assayed by CD31 and α-SMA staining on sections of these tumours). The immune landscape of the TH-MYCN tumour following JQ1 treatment showed an increased percentage of CD8+PD-1+, CD4+PD-1+, and Treg PD-1+ cells compared to controls, although it did not affect immune cell infiltration to the TME. Interestingly, anti-PD-1 and JQ1 treatment reduced TH-MYCN tumour volumes on days 8 and 15 compared to controls. In conclusion, these studies linked small molecule inhibitors with the tumour TME in NB and immune cells, hypoxia, and blood vessel architecture that were covered earlier in this study [27].

The inhibition of NKG2DL by antibodies led to increased CD8+ T cells expressing IFN-γ, Ki-67, and CD107 markers, suggesting that ligand inhibition in this case can reverse immunosuppression and counter a tumour-friendly TME [28]. In agreement with the study, the immunosuppressive nature of the NB TME and the role of HMGB1 proteins in inducing Treg cell differentiation and contributing to immunosuppression were studied. The SK-N-SH secretome contained high levels of HMGB1 compared to other NB cells, including SK-N-AS cells [63]. The coculture of human PBMCs with SK-N-SH NB cells for 4 days led to CD4+ CD25+ Foxp3+ Treg differentiation, while the blockage of HMGB1 using anti-HMGB1 monoclonal antibodies could suppress Treg differentiation. This result was also obtained when exposing human PBMCs to the supernatants of SK-N-SH cell culture. Interestingly, the authors studied 498 NB patients’ expression data from the GSE49711 dataset and showed that 11% of patients with overexpressed HMGB1 also showed a higher risk of disease progression and relapse. Overall, this suggested that the blockage of proteins linked to immunosuppression in the TME could be a viable therapeutic strategy to treat NB and improve patient prognosis [63].

Overall, this review investigated the relationship between multiple factors, including the ECM, immune cell infiltration, proteins, RNA, EVs, NB identities, and treatment options and strategies targeting the reversal of immunosuppression that impacted the TME and NB interaction and thereby affected NB biology, prognoses, progression, and treatment.

## Data Availability

Not appliable.

## Abbreviations

ADCC:	Antibody-dependent cell-mediated cytotoxicity
α-SMA:	α smooth muscle actin
B3GALT4:	Beta-1,3-galactosyltransferase
BM-MSCs:	Bone marrow-derived mesenchymal stem cells
CAFs:	Cancer-associated fibroblasts
CAR:	Chimeric antigen receptor
CRCs:	Core regulatory circuitries
ECM:	Extracellular matrix
EVs:	Extracellular vesicles
Fab-CHP:	FAM conjugated to collagen hybridising peptides
FAK:	Focal adhesion kinase
IDRFs:	Image-defined risk factors
IHC:	Immunohistochemistry
INRG:	International Neuroblastoma Risk Group
INRGSS:	International Neuroblastoma Risk Group Staging System
INSS:	International Neuroblastoma Staging System
IRF9:	Interferon regulatory factor 9
NK:	Natural killer
NMT:	Noradrenergic-to-mesenchymal transition
MDSCs:	Myeloid-derived regulatory cells
MS:	Metastatic special
PBMC:	Peripheral blood mononuclear cells
PD-1:	Programmed cell death protein 1
PDX:	Patient-derived xenograft
PMN:	Premetastatic niche
PTBP2:	Polypyrimidine tract binding protein 2
TAMs:	Tumour-associated macrophages
TEVs:	Tumour extracellular vesicles
TFs:	Transcription factors
TME:	Tumour microenvironment
Treg:	Regulatory T cells
TrkA:	Neurotrophin receptor tropomyosin-related kinase
RA:	Retinoic acid
SCPs:	Schwann cell precursors
SEMA3A:	Semaphorin 3A
SHMT2:	Serine hydroxymethyltransferase 2
